# Variability and error in measurement of infant formula powder and water: an experimental study

**DOI:** 10.3389/fnut.2024.1385496

**Published:** 2024-08-07

**Authors:** Richard R. Rosenkranz, Ana Gonzalez-Alvarez, Chris Acosta, Andrew Hooyman, Jose R. Hidalgo, CindyRomina Ballesteros-Paniagua, Sara K. Rosenkranz

**Affiliations:** ^1^Department of Kinesiology and Nutrition Sciences, University of Nevada, Las Vegas, Las Vegas, NV, United States; ^2^Department of Food, Nutrition, Dietetics, and Health, Kansas State University, Manhattan, KS, United States; ^3^Department Social and Behavioral Health, School of Public Health, University of Nevada, Las Vegas, NV, United States

**Keywords:** Infant, formula, feeding, breastfeeding, bottle feeding, over-feeding, measurement, home nutrition support

## Abstract

**Introduction:**

Formula feeding is the only viable nutrition alternative for infants 0–6mos who cannot breastfeed. Among the drawbacks of formula feeding, however, is potential dilution or concentration errors in the formula during preparation that may lead to infant health issues. The present study aimed to investigate the accuracy of caregiver measurements as they prepared infant formula under multiple conditions, compared with manufacturer specifications.

**Methods:**

A diverse sample of caregivers (*N* = 84) participated in this cross-over experimental study. Participants hand-scooped infant formula powder and poured water to prepare 4oz. and 7oz. feedings, using both a standardized set of infant formula products and participants’ own products. Linear mixed effects models were used to estimate fixed effects of target amount (4oz. versus 7oz) and products (participant versus researcher) on mean absolute percent error (MAPE) of measurement.

**Results:**

Across all conditions MAPE was significantly greater for measuring powder than for water (9.0% vs. 4.4%; *p* < 0.001) with a combined powder and water MAPE at 13.0%. Greater measurement error was associated with the odd-sized 7oz. preparation and participants’ own products.

**Discussion:**

We observed considerable variability and substantial error during infant formula preparation, particularly for hand-scooping of powder, which tended toward higher values than the theoretical gold standard.

## 1 Introduction

The first 1,000 days of life are a critical period for the foundation of children’s life-long health, learning, and wellbeing (1). Malnutrition during those first 1,000 days of life is a serious public health problem that can have devastating consequences for health, development, and productivity over the life course (2). This public health problem can manifest in stunting, wasting, micronutrient deficiencies, overweight or underweight status. Malnutrition can also result in impaired cognitive development and learning, leading to lower academic achievement and lower earning potential in adulthood.

Breast milk is tailored to infants’ nutritional needs, containing the right balance of proteins, fats, carbohydrates, vitamins, and minerals, along with antibodies and other immune factors that help protect babies from infection and disease (3). The World Health Organization recommends exclusive breastfeeding for infants’ first 6 months and then continued breastfeeding for up to 2 years or more alongside complementary foods. Breast milk presents little to no risk of foodborne illness when delivered directly through breastfeeding. Many infants are formula fed, due to a range of reasons (4). According to the Centers for Disease Control and Prevention (CDC), in 2019, 83% of infants born within the United States started out receiving some breast milk (5). At the age of 6 months, however, only 56% of infants were receiving any breast milk, and only 25% were receiving breast milk exclusively (5). Beyond the U.S., only 37% of children aged under 6 months are exclusively breastfed in low- and middle-income countries (6). Sales of infant formula are growing worldwide, which the World Health Organization attributes to aggressive and often unethical marketing of products in a $55B industry (7).

Infant formula is uniquely critical as the sole nutritional source for infants who are not breastfed as it replaces breastmilk—a complex biological tissue providing for infants’ needs. Consequently, the formula’s composition is vital, requiring a precise balance of nutrients to support rapid early development. The preparation of formula, particularly the accurate reconstitution of powdered or concentrated forms, is equally crucial (8, 9). Improper mixing can lead to nutritional imbalances that pose significant health risks, such as malnutrition or hypernatremia. Under-diluting infant formula can result in short-term health problems such as hypernatremic dehydration, gastroenteritis, and other digestive problems or long-term excessive weight gain and obesity (8–10). Over-diluting infant formula could also lead to serious health problems for babies, including, diarrhea, water intoxication, nutrient deficiencies (potentially manifesting as stunting, wasting, or underweight), and even death (8–10).

Previous studies have assessed the measurement accuracy of caregivers’ infant formula preparations in relation to bottle characteristics, package instructions, caregiver experience, and target amount (11–16). In a systematic review published in 2003 by Renfrew et al. (10), all five of the included studies showed substantial problems achieving the proper concentration of infant formula, with a tendency toward over-concentration (under-dilution) of formula. A more recent laboratory study of caregivers and non-caregivers (12) showed consistent over-dispensation of powdered formula across 2oz., 4oz., 6oz., and 8oz. preparations, equivalent to 11% excess calories per bottle (under-dilution). Few studies, however, have separately investigated the variability of hand-scooping infant formula powder and pouring water: 1) For both caregivers and a trained measurer; 2) For the caregiver’s own personal formula products and a standardized set of products; 3) In relation to a variety of demographic variables. The purpose of the present study was to determine to what degree accurate powder and water measurements are made in accordance with the formula manufacturer specifications by caregivers and an trained measurer when preparing infant formula for feeding under multiple conditions. The study also aimed to determine the influences on the variability in hand scooping and liquid measurement during the caregivers’ preparation of powdered infant formula.

## 2 Materials and methods

### 2.1 Study design and setting

This study employed a cross-over experimental design, wherein each participant measured formula powder and water under multiple conditions. Separately, a trained research assistant measured infant formula powder and water 30 times each, under multiple highly controlled conditions. The research was approved by Institutional Review Boards for data collection in two locations: The Physical Activity and Nutrition Clinical Research Consortium (PAN-CRC) within Lafene Health Center at Kansas State University; and The Behavioral Nutrition and Physical Activity laboratory at the University of Nevada, Las Vegas.

Participants were contacted and recruited through various means, mainly through social media (particularly Facebook groups of mothers), university announcements, email, text, and referral. Interested caregivers emailed, texted, or called the primary investigator, were screened for eligibility criteria, provided with a general study description, and given an opportunity to ask questions before scheduling their study appointments. Eligibility criteria required participants to be: at least 18 years old; a mother or caregiver who was currently formula feeding an infant; fluent in English or Spanish; and willing to attend a research appointment in person for about 30–45 min.

### 2.2 Participants

A total of 84 participants were included in this study, 17 from Kansas and 67 from Nevada. Caregiver characteristics are summarized in Table 1. A majority of participants from each state had no affiliation with the university but resided in the local community. The mean age of the participants was 31.1 years (SD = 7.36), and the mean age of participants’ infants was 7.2 months (SD = 5.16). A large majority of participants (*n* = 71) identified themselves as the infant’s primary caregiver and 30 participants indicated that they were simultaneously breastfeeding and supplementing with infant formula. More than one-third of participants (*n* = 32) were enrolled in the Special Supplemental Nutrition Program Women, Infants, and Children (WIC). Regarding the duration of breastfeeding, 39 participants reported never having breastfed their infant or breastfeeding for less than 1 month, 27 participants had breastfed for 1 to 5 months, and 18 participants had breastfed for 6 months or more. About one-third of participants (*n* = 29) reported bottle-feeding their infants one to three times daily, while nearly two-thirds (*n* = 54 participants) fed their infants four times or more daily. Only 5 participants reported pre-paring 2 ounces or less per feeding, while half (*n* = 42) reported preparing 3 to 5 ounces per feeding, and the remaining participants (*n* = 35) reported preparing 6 ounces or more per feeding or did not report the amount (*n* = 2). The sample was racially diverse, with a small majority (*n* = 44) of participants reporting their race as White. The remainder reported being Black or African American (*n* = 7), American Indian or Alaska Native (*n* = 1), Asian (*n* = 4), Mixed/other Race (*n* = 27), or did not report (*n* = 2). A sizable minority (*n* = 39) reported Hispanic ethnicity. There was also ample educational and household income diversity among participants (see Table 1).

**Table 1:** Participant demographics (*N* = 84).

Caregiver characteristic	Mean	SD	Not reporting (*n*)
Age (years)	31.1y	7.36y	5
Infant’s age (months)	7.2 m	5.16 m	1

Caregiver characteristic	Subgroup (*n*)	% of sample	Not reporting (*n*)
Main caregiver (yes)	71	84.5	1
WIC participation (yes)	32	38.1	1
Currently smoking/vaping (yes)	16	19.0	1
Currently also breastfeeding (yes)	30	35.7	0
Hispanic (yes)	39	46.4	1
Race			2
White	44	52.4	
Black or African American	7	8.3	
American Indian or Alaska Native	1	1.2	
Asian	4	4.8	
Mixed/other race	27	32.1	
Education			1
Less than high school graduate	5	6.0	
High school graduate	19	22.6	
Some college or associate’s degree	31	36.9	
Bachelor’s degree or higher	29	34.5	
Household income ($/year)			3
Less than $35 K	22	26.2	
From $35 K to <$100 K	38	45.2	
$100 K or higher	21	25.0	
Breastfeeding duration (months)			0
Never or less than 1 month	39	46.4	
From 1 to 5 months	27	32.1	
6 months or more	18	21.4	
Frequency of bottle feeding			1
One to three times daily	29	34.5	
4 times or more daily	54	64.3	
Amount of formula used (oz)			3
2 ounces or less per feeding	5	6.0	
3 to 5 ounces per feeding	42	50.0	
6 ounces or more	35	41.7	

### 2.3 Measures and procedures

#### 2.3.1 Bottles and formula

Participants were instructed to bring up to five of their own baby bottles with them to the laboratory, including an 8oz. bottle. They were also asked to bring a container of their usual powdered baby formula, complete with a scoop and intact label. In situations where participants did not bring their personal formula preparation products (hereafter referred to as products), researchers provided extras for them to use (Similac Advance infant formula and Evenflo Feeding Classic Clear Plastic Baby Bottles). Research assistants observed and recorded details of participants’ bottles and formula powder, noting relevant information such as size, style, number of grams per scoop for formula, color, material, and volume scale for bottles. They also gathered information about the types and brands of formula and bottles typically used, including whether those differed from the products they brought with them. All bottles were marked with an identification sticker and then weighed to the one-hundredth of a gram while empty and dry, without any nipple or collar, on a calibrated Bonvoisin Lab Scale 5,000 g x 0.01 g high precision electronic analytical balance.

#### 2.3.2 Demographic questionnaire

Participants completed a demographic questionnaire, providing information about themselves that included age, socioeconomic status, infant birthday, household characteristics, breastfeeding duration, and the amount and frequency of bottle feeding. Summary data are presented in Table 1.

#### 2.3.3 Infant formula preparation and measurement

Participants were first asked to describe their typical steps for preparing infant formula. Research assistants recorded notes about these preparation steps, including any additional preparation products that were mentioned. Scooping of infant formula powder and pouring of water was not performed until after participants described their typical steps. Next, participants were instructed to take their own bottles and powder formula, then follow their typical steps of scooping powder and pouring water. Hand sanitizer and a leveling utensil were available within participants’ reach. They were first asked to scoop infant formula powder as they normally would if they were preparing a 4oz. bottle. Researchers took a picture of the first scoop of powder, just before it was added to the bottle (see Supplementary Figure S1 for a selection of images). Next, participants were asked to scoop infant formula for preparation of a 7oz. bottle. This odd size (7oz) was specifically chosen because older infants may consume more than 6 ounces during a feeding and caregivers sometimes must choose between discarding costly unused formula, keeping leftover formula at the risk of contamination, or having to prepare additional formula for an unsated baby. We hypothesized that more errors would result from an odd-size feeding because most infant formula scoops each hold enough powder when full and leveled for 2oz. of prepared formula. For both 4oz. and 7oz. preparation, the steps were carefully documented, including spillage details, whether the formula was leveled or packed, plus any relevant notes about the process.

After powder was scooped and added to the bottle for either a 4oz. or 7oz. feeding, research assistants obtained weights from the Bonvoisin Lab Scale, meticulously recording them on a data sheet. Later, the previously recorded bottle dry weight was subtracted from the total bottle and powder weight to obtain the mass of formula powder that was scooped and poured into the bottle. Because this study was conducted during a time where there were formula shortages, once finished weighing was completed, researchers asked permission from the participants to return the measured dry powder formula back to the participant’s container, to prevent wasting valuable formula.

After measuring the powder formula, participants were asked to pour water as if they were preparing for a feeding. Although some caregivers reported using hot or boiling water in their usual preparations, the basic process of adding water to a target volume in the bottle is uniform. So, we standardized the water measurements for convenience by making room-temperature water available in a pitcher for participants to pour. Participants were asked to pour water first for a 4oz. feeding, and then for a 7oz. feeding, simulating their usual practices. Research assistants obtained weights of poured water within bottles from the Bonvoisin Lab Scale, recording them on a data sheet and subtracting empty bottle weight as described above.

Once finished with scooping powder and pouring water for the 4oz. and 7oz. feeding by using their own products (formula powder and bottles), we repeated the entire measuring process after providing participants with a standardized set of products. Those products consisted of researcher bottles and infant formula powder (Love and Care Infant Formula Powder and a second set of Evenflo Feeding Classic Clear Plastic Baby Bottles). Participants were invited to familiarize themselves with the bottles and infant formula powder instructions if they felt it necessary to do so.

After completing the scooping and pouring tasks using both their own products and those of the researchers, participants completed the second portion of a questionnaire related to their usual baby feeding practices. Those data are intended for a separate investigation and are not relevant to the present study. Before participants finished their research appointment, they were invited to review an infant formula preparation best practices information sheet from the Centers for Disease Control and Prevention or to watch a video presenting infant formula preparation best practices from WIC. While participants reviewed those best practices materials, research assistants checked the completeness of data from questionnaires and observations. Participants were compensated for their time and travel costs with a $100 Amazon gift card.

#### 2.3.4 Gold standard

The weights, in grams, that were listed on the package label for each type of infant formula powder (either brought in by the caregivers or provided by the researchers) served as the theoretical gold standard for comparison. For the Love and Care formula, the theoretical value was 8.8 g per scoop to prepare 2oz. of formula, so 17.6 g for a 4oz. feeding and 30.8 g for a 7oz. feeding. For water at room temperature, a theoretical value of 29.57 grams per 1oz. served as the basis for the gold standard values of 118.29 g for 4oz. and 207.015 g for 7oz. (17).

After extensive training and measurement protocol development—consistent with the formula preparation instructions from the manufacturer—trained research assistant measurements were also evaluated as a best case scenario for real-world measurement. Separate from the research participant appointments, the research assistant performed a series of powder and water measurements under varying highly controlled conditions, according to the standardized measurement protocol. All trained research assistant measurements used the Love and Care Infant Formula powder, Evenflo Feeding Classic Clear Plastic bottles, and the water pitcher to conduct these careful measurements. Both powder and water were carefully measured, weighed, and recorded 30 times each for the 4oz. and 7oz. preparations. The research assistant scooped from the canister and leveled powder within the scoop using a straight-edged utensil before carefully adding the powder to a standard previously weighed bottle. Two level scoops were added for the 4oz. preparation and 3 scoops for the 7oz. preparation. For the last half-scoop of powder needed to prepare a 7oz. feeding (3.5 scoops), the research assistant used and leveled a separate half-size scoop. In addition to 30 separate measurements that aligned with manufacturer specifications, they also scooped and packed formula powder 30 times for both 4oz. and 7oz. feedings to create a compacted measurement because some caregivers have been observed compacting the formula within the scoop. Last, the research assistant poured room temperature water at eye level until the bottom of the meniscus was at 4oz. and then at 7oz. within a standard previously weighed bottle. Each of the 30 pouring measurements for both 4oz. and 7oz. was carefully weighed and recorded using the Bonvoisin Lab Scale. The mean of each set of 30 non-compacted measurements served as the trained research assistant measurement standard.

### 2.4 Statistical analysis

For each participant’s measurement of 4oz. and 7oz. preparations, the mean absolute percent error (MAPE) was calculated by dividing the participant measurement in grams by the theoretical gold standard, subtracting 1.0, and then multiplying the absolute value of the result by 100. Initial examination of the formula and water MAPE demonstrated significant rightward skew; thus, they were log-transformed for parametric procedures. MAPE was also calculated for each set of combined powder and water measurements for 4oz. and 7oz. preparations, reflective of what error would be for each bottle of prepared formula.

Separate linear mixed effects models, one for formula and one for water, were used to estimate the primary fixed effects of the targeted amount (4oz. versus 7oz) and products used (caregiver’s own versus researchers’ standardized set) on mean absolute percent error (MAPE) of measurement. Additional fixed effects used as statistical control variables comprised current breastfeeding status (yes/no), frequency of bottle feeding per day (ordinal 1–3), amount of bottle feeding (ounce ranges, ordinal 1–4), primary caregiver status (yes/no), age of the infant (interval), and scoop type (half- or full-size, only used for models of formula powder MAPE) with random intercepts of participant.

A generalized linear mixed effects model with binomial link function was used to examine the primary fixed effects of targeted amount (4oz. versus 7oz) and bottle type (participant versus researcher) on the odds of the combination of powder and water MAPE being ≥10% for each set of measurements. Similar to the models above, additional fixed effects that were used as statistical control variables comprised current breastfeeding status, frequency of bottle feeding per day, amount of bottle feeding, primary caregiver status, age of the infant, and scoop type, with random intercepts of participant.

All fixed effect coefficients, and their respective 95% confidence intervals, were back-transformed using exponentiation for better interpretability. This resulted in an estimated coefficient that represents the multiplicative factor for every one-unit increase in the independent variable. For example, a back-transformed coefficient equal to 1.2 represents that for every one-unit increase in our independent variable, there was an increase in the dependent variable by a factor of 1.2, or 20% more than the original value.

A linear mixed effects model with a primary fixed effect of substance was used to examine differences in MAPE between formula versus water. Again, additional fixed effects included as statistical control variables were current breastfeeding status, frequency of bottle feeding per day, amount of bottle feeding, primary caregiver status, age of the infant, and scoop type, with random intercepts of participant.

One-sample t-tests were used to compare the trained research assistant measurements to the theoretical gold standard. Thus, this test was used to assess the presence or absence of a significant deviation between what the research assistant could carefully measure and what the infant formula label indicated. The family-wise alpha level for multiple comparisons in this study was set at 0.05, ensuring a controlled overall type I error rate across the various statistical tests conducted.

## 3 Results

### 3.1 Descriptive statistics

Across 84 participants and conditions, the infant formula powder MAPE was 9.03% (SD = 11.84%). This indicates that on average, measurements differed from the theoretical gold standard—either over or under—by more than 9%. Actual percentage of error ranged from −69.6 to +78.8% for powder. Across participants and conditions, the water MAPE was 4.45% (SD = 8.80%), indicating a difference of more than 4% from the theoretical gold standard. Actual percentage of error ranged from −54.3 to +20.0% for water. The combined MAPE for measuring powder and water was 13.04% (SD = 19.55%), which means that the prepared formula would have differed from the theoretical gold standard by more than 13%. Actual percentage of error ranged from −69.4 to +125.0% for the combination of powder and water.

Because indicators of central tendency such as MAPE often do not convey the prevalence of substantial errors in measurement, the proportion of measurements that differed from the theoretical gold standard by 10% or more was tallied and also used in binomial link function analyses (see below). While there is not consensus within the literature on what constitutes large errors, Altazan et al. (12) modeled problematic implications for infant health from 10 to 11% over-dispensation of formula. Across participants and conditions, approximately one-fourth of infant formula powder measurements (25.6%) and one-fifteenth of water measurements (6.5%) showed substantial error. More than three-tenths of the combined powder and water measurements showed substantial error (30.6%). Last, two-thirds of participants (66.7%) made at least one measurement error that was 10% or greater.

### 3.2 Effects of substance, targeted amount, and products used

**Figure 1:**
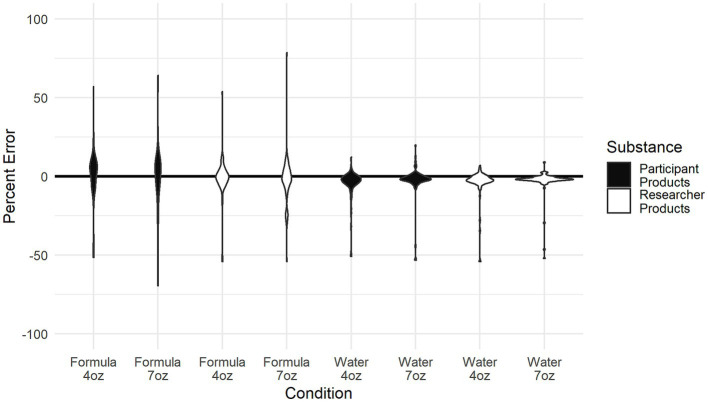
Depiction of the variability in the mean average percent error of infant formula measurement by powder, water, size, and whose products were used by participating caregivers.

Figure 1 depicts variability in the MAPE of infant formula measurement by powder, water, size, and products used by participating caregivers. In linear mixed effects models that mutually adjusted for relevant caregiver characteristics (breastfeeding status, frequency of bottle feeding per day, amount of bottle feeding, primary caregiver status, age of the infant, and scoop type), significant differences in error were observed between measurements of infant formula powder and measurements of water (*β*Water =0.40, 95% CI = [0.33; 0.48], *p* < 0.001). This result indicates that—across the targeted amount and products used (caregiver’s own personal formula products or standardized set)—the participants’ measurements showed substantially less error for water, compared to infant formula powder.

Complete model results for powder MAPE can be viewed in Table 2. In the linear mixed-effects models that mutually adjusted for relevant caregiver characteristics (breastfeeding status, frequency of bottle feeding per day, amount of bottle feeding, primary caregiver status, age of the infant, and scoop type), there was a significant effect of targeted amount (4oz. vs. 7oz) on infant formula powder MAPE (*β*7ozP = 1.36, 95% CI = [1.09; 1.69], *p* = 0.006). This result indicates that powder MAPE during the preparation of a seven-ounce bottle was 36% higher than powder MAPE for the four-ounce bottle. In the linear mixed effects model, there was a significant effect of products used (caregiver’s own versus standardized set) on powder MAPE (*β*participantP = 1.77, 95% CI = [1.43; 2.21], *p* < 0.001). Specifically, there was 77% greater powder MAPE for the caregiver’s products, compared to the standardized set. There was no significant interaction of targeted amount by products used on powder MAPE, indicating that the error observed between infant formula powder targeted amount (4oz. versus 7oz) did not vary by whose products were used.

**Table 2:** Effects on mean average percent error of infant formula powder measurement.

Variable name	MAPE_log_ estimate	SE	MAPE back-transformed estimate	DF	*t*-value	*p*-value
Intercept	2.22	0.52	9.24	241	4.24	0.000
Caregiver’s own products^*^	0.57	0.11	1.77	241	5.18	0.000
Seven-ounce targeted amount^a^	0.31	0.11	1.36	241	2.79	0.006
Currently breastfeeding^b^	−0.13	0.04	0.88	74	−3.43	0.001
Frequency of bottle feeding^c^	−0.03	0.06	0.97	74	−0.61	0.547
Amount of bottle feeding^d^	−0.33	0.14	0.72	74	−2.37	0.020
Half-size scoop^e^	0.20	0.11	1.22	74	1.86	0.067
Primary caregiver^f^	−0.01	0.24	0.99	74	−0.06	0.956
Age of the infant^g^	−0.02	0.20	0.98	74	−0.11	0.913

^*^Reference is researchers’ standardized set of products.

a Reference is four-ounce target.

b Reference is not currently breastfeeding.

c Ordinal scale of frequency 1–3.

d Ordinal scale of amount 1–4.

e Reference is full-size scoop.

f Reference is not the primary caregiver.

g Continuous variable with months as units.

Complete model results for water MAPE can be viewed in Table 3. In the linear mixed-effects models that mutually adjusted for relevant caregiver characteristics, there was no significant effect of targeted amount on water MAPE (*β*7ozW = 0.85, 95% CI = [0.66; 1.10], *p* = 0.22). Also, there was no significant effect of products used on water MAPE (*β*participantW = 0.89, 95% CI = [0.68; 1.15], *p* = 0.36). There was no significant interaction of products used by targeted amount on water MAPE. This indicates that the level of error observed between products used did not vary due to water targeted amount (4oz. versus 7oz).

**Table 3:** Effects on mean average percent error of water measurement.

Variable name	MAPE_log_ estimate	SE	MAPE back-transformed estimate	DF	*t*-value	*p*-value
Intercept	1.22	0.58	3.37	241	2.09	0.038
Caregiver’s own products^*^	−0.12	0.13	0.89	241	−0.92	0.359
Seven-ounce targeted amount^a^	−0.16	0.13	0.85	241	−1.23	0.219
Currently breastfeeding^b^	0.00	0.05	1.00	75	0.01	0.991
Frequency of bottle feeding^c^	−0.06	0.07	0.94	75	−0.88	0.380
Amount of bottle feeding^d^	−0.16	0.16	0.85	75	−1.00	0.322
Primary caregiver^e^	0.07	0.28	1.08	75	0.27	0.792
Age of the infant^f^	0.12	0.23	1.12	75	0.51	0.611

^*^Reference is researchers’ standardized set of products.

a Reference is four-ounce target.

b Reference is not currently breastfeeding.

c Ordinal scale of frequency 1–3.

d Ordinal scale of amount 1–4.

e Reference is not the primary caregiver.

f Continuous variable with months as units.

### 3.3 Modeling large errors

As summarized in Table 4, the binomial link function model was used to investigate effects on large errors (MAPE ≥10%) in the combined powder and water measurement. The present study’s results showed that when statistically controlling for other variables in the model (i.e., breastfeeding status, frequency of bottle feeding per day, amount of bottle feeding, primary caregiver status, age of the infant, and scoop type), there was a significant effect of products used (caregiver’s own versus standardized set) on the likelihood of large error (OR = 4.23, 95% CI = [2.23; 8.04], *p* < 0.001). Specifically, participants were about four times more likely to measure amounts of formula powder and water that were at least 10% off target when using their own products, compared to the standardized set.

**Table 4:** Effects on large errors (≥10%) in the combination of powder and water measurement.

Variable name	Odds ratio	Lower 95% CI	Upper 95% CI	*p*-value
Intercept	2.28	0.07	77.49	0.647
Caregiver’s own products^*^	4.23	2.23	8.04	0.000
Seven-ounce targeted amount^a^	1.25	0.69	2.25	0.455
Currently breastfeeding^b^	0.74	0.53	1.04	0.085
Frequency of bottle feeding^c^	0.71	0.31	1.59	0.404
Amount of bottle feeding^d^	1.89	0.99	3.59	0.052
Half-size scoop^e^	0.15	0.01	1.89	0.142
Primary caregiver^f^	0.72	0.19	2.80	0.639
Age of the infant^g^	0.56	0.17	1.83	0.341

^*^Reference is researchers’ standardized set of products.

a Reference is four-ounce target.

b Reference is not currently breastfeeding.

c Ordinal scale of frequency 1–3.

d Ordinal scale of amount 1–4.

e References is full-size scoop.

f Reference is not primary caregiver.

g Continuous variable with months as units.

### 3.4 Trained research assistant measurement error

**Figure 2:**
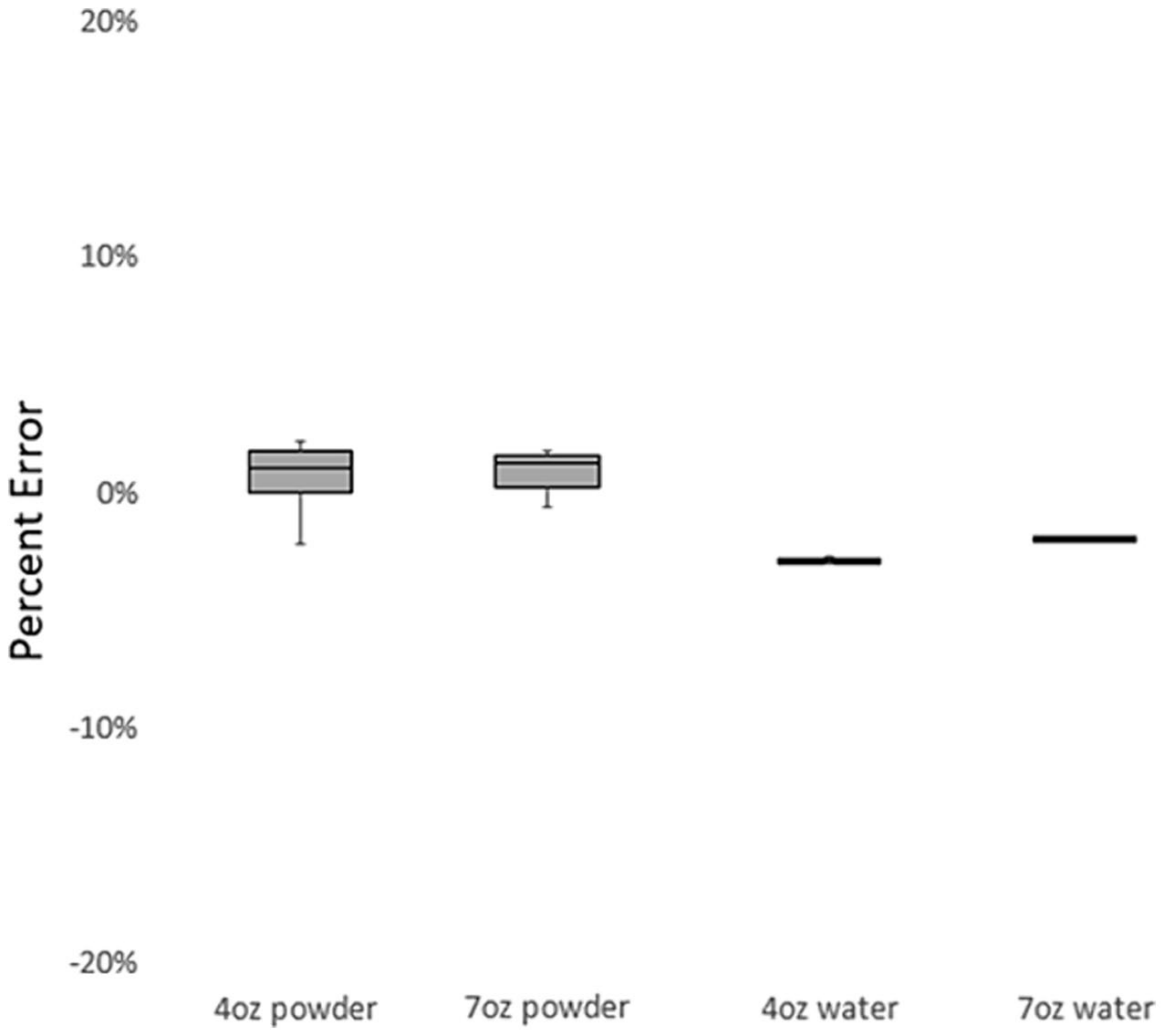
Trained research assistant measures of powder and water by condition.

Figure 2 displays the percentage of error in measurements performed by the trained research assistant under various conditions. Low percentage of error values were seen for the 4oz. powder (1.05%), 7oz. powder (1.27%), 4oz. water (−2.70%), and 7oz. water (−1.76%).

Table 5 presents comparisons between the theoretical gold standard (values expected from infant formula label) and the trained research assistant measurements. For all conditions, the trained research assistant measurements significantly differed from the theoretical gold standard (*p* < 0.001).

**Table 5:** Theoretical vs. trained research assistant measures of powder and water.

Condition	Expected^a^ value (g)	Mean observed^b^ Value (g)	Lower 95% CI	Upper 95% CI	*t*-value	*p*-value
4oz. powder	17.6	17.47	17.40	17.54	−3.547	0.001
7oz. powder	30.8	30.49	30.41	30.57	−7.449	< 0.001
4oz. water	118.3	115.11	115.08	115.14	−180.632	< 0.001
7oz. water	207.0	203.41	203.34	203.48	−101.531	< 0.001

a Expected value represents the theoretical gold standard derived from powder formula label and known properties of water.

b Mean observed value represents the central tendency of the practical gold standard derived from trained research assistant measures.

Of note, the 4oz. compacted powder (17.87%) and 7oz. compacted powder (18.39%) had the highest error among measurements by the trained research assistant. For both 4oz. (*t* = 283.9, *p* < 0.001) and 7oz. (*t* = 657.5, *p* < 0.001), the observed measurement values were significantly different from the expected values.

## 4 Discussion

Our study sought to determine to what degree accurate measurements are made in accordance with the formula manufacturer specifications mainly by caregivers—but also by a trained measurer—when they prepare infant formula for feeding under multiple conditions. Although there was error both when participants hand-scooped infant formula and added it to their bottles (9%) and when participants poured water into bottles (4.4%), the largest magnitude of error was from the combined powder and water preparations (13%). There was wide variability in where participants made errors. Some participants were consistently higher or lower than the gold standard for both powder and water, which effectively would minimize the error for the combination. The general tendency, however, was for participants to over-dispense powdered formula and to underpour water. Contributing to the underpouring of water was approximately 2–3% error that can be attributed to inaccurate markings on the Evenflo bottles, a finding revealed by the differences between the trained measurers and the gold standard. This tendency to over-dispense powder and underpour water exacerbated each of the individual component errors to result in a formula that was an average of 13% more concentrated than it should have been, surpassing the individual error contributions from powder or water alone. Put into caloric terms, a 13% error would add approximately 10kcals to each 4oz. feeding or 18kcals to each 7oz. feeding. Since most participants were feeding their infants four times or more daily, many infants could be receiving substantial amounts of excess energy each day.

Photographs and observations that were made during this study revealed that many caregivers: failed to level a heaping scoop carefully; sometimes failed to fill the scoop; and often spilled some powder while adding powder to the bottle. Occasionally, caregivers compacted the formula by scooping powder into the side of the can. In a few cases, caregivers seemed to lose track of the number of scoops and either missed one or added an extra scoop of powdered formula. In contrast, the trained measurements made by the trained research assistant achieved a closer approximation of the target and theoretical gold standard, but still showed significant differences from the intended values that would result in under-diluted or over-concentrated formula.

Renfrew et al. (10) described a general tendency reported within the scientific literature for under-dilution or over-concentration of formula. The present study supported that general tendency within the literature while investigating specific drivers of variability. Our results showed that the contribution of error from formula powder measurement was approximately twice the magnitude of error from water measurement. Given the complexity of the task of scooping powder from a can without compacting the powder, leveling the scooped powder, and then transferring it from the scoop into the bottle, there are multiple places where errors may accumulate. The present study was not equipped to investigate the individual contributions of each separate stage of powder measurement (scooping, leveling, transferring), but future studies should determine which of them contributes most to the total error. For the combined error of powder and water, we found an average of approximately 13% under-dilution (over-concentration) in the present study, which is slightly higher than that reported by Altazan et al. (12) although their study did not separately assess water measurement. Notably, that 11% was seen in a study sample of both caregivers and non-caregivers, while our sample of all caregivers did no better, without any significant difference between primary caregivers and non-primary caregivers. Thus, being a primary caregiver does not appear to confer any particular advantage to infant formula measurement accuracy.

Contrary to what may have been expected, demographic variables were generally not significantly explanatory of error in measurements. There was some evidence that those who were currently breastfeeding and those feeding larger amounts made smaller errors during the measurement of formula powder, but those factors were not significant for water, nor for large errors in the combination of powder and water. Furthermore, frequency of bottle feeding, scoop size, and age of infant were not significant; neither were education nor income (data not shown). Overall, it appeared that error was not connected to any particular demographic but was quite widespread across the sample. Indeed, one-quarter of the sample measurements had 10% or greater error for formula powder, almost one-third had ≥10% error for the combination of powder and water, and two-thirds of participants made at least one large error of ≥10% in their powder and/or water measurements.

It is important to remember the potential health implications of mismeasuring infant formula. Over the short to medium term, under-diluting infant formula could lead to hypernatremic dehydration, gastroenteritis, and other digestive problems (8). Over the long term, under-dilution at a level such as that shown in our results could lead to excessive weight gain and obesity (12). Indeed, global concern over increasing child obesity rates highlights the impact of infant feeding practices. Research shows that infants consuming powdered formula, possibly due to reconstitution errors, ingest more calories and have increased fat deposition, as compared to ready-to-feed formula (18). On the other hand, some caregivers in our study measured powder and water in a way that would over-dilute infant formula, potentially leading to short-term health problems such as diarrhea and water intoxication (8). Over the long term, over-dilution could lead to nutrient deficiencies or even death (10).

The present study advances the current body of evidence, showing a substantial difference in error from formula powder measurement versus error from water measurement, and how the divergent directions of those errors combined to exacerbate under-dilution. Ostensibly, water measurement poses a less complex measurement problem, although extant literature has identified how even health professionals can have difficulty with liquid measurement accuracy (19). Furthermore, baby bottle manufacturers may contribute to measurement difficulties with difficult-to-read markings, irregularly shaped bottles, opaque materials, or inaccurate markings on the bottle constituting barriers to preparing appropriate concentrations of infant formula (13–15). One study (15) found that baby bottles purchased in Australia and labeled as meeting the European Standard for infant feeding bottles (including a volume marker accuracy requirement) were no less likely to be accurate than bottles that were not labeled as meeting the European Standard. Although having standards for accurate volume markings is important, such standards must be enforced to benefit the consumer.

The most unexpected finding in the present study was that the caregivers made larger errors with their own products, as compared to with the standard researcher set that was provided. That is, caregivers made greater errors in powder measurement and were more likely to make substantial error (>10%) in both preparation of powder and water when they were asked to prepare formula with their own powders, scoops, and bottles versus a presumably unfamiliar set of products. It is unclear why this occurred but it is unlikely due to an order effect as the order in which caregivers used each set of products was variable during their visit. One potential explanation may come from habit theory and dual process theory (20). According to those theories, frequently repeated actions may become automatic, rather than requiring focused attention and conscious deliberation. When caregivers used their own products in the preparation of formula, they may have been operating according to well-established habitual action patterns. When asked to use unfamiliar infant formulas, scoops, and bottles, caregivers may have moved from system 1 to system 2 thinking, bringing conscious awareness, deliberation, and a focused attention to the task of measuring accurately under those conditions. Related research has shown how the absence of attentional focus through maternal distraction can contribute to feeding problems (21). Although large errors were prevalent for the condition where caregivers were confronted with unfamiliar products, it was significantly less error-prone than when using their own familiar products.

The present study is limited in terms of a modest sample size and a short-term investigation of error during the measurement of formula powder and water. It is possible that the findings from this sample do not generalize to the wider population despite the diversity of participants and concordance with extant literature. The study is also limited in ecological validity in that participants were asked to measure powder separately before water, and to do so in a laboratory at a university; that setting may be very different from where they usually prepare infant formula. Such differences could contribute to greater errors than normal. In contrast, participants who are being observed within a research study are often subject to the Hawthorne effect or demand characteristics that may have them acting more carefully or productively than if not being observed. Strengths of this study included the rigorous study protocol, multiple careful measurements made on a precision scale, investigation of many putative drivers of error, and the diversity of the sample.

## 5 Conclusion

In this study, participating caregivers demonstrated considerable variability and substantial error during infant formula preparation, particularly for hand-scooping of powder, which tended toward higher values than the theoretical gold standard. Greater measurement error was associated with the odd-sized 7oz. preparation and—unexpectedly—with the participants’ own products. In combination, errors from powder and water often compounded, generally toward under-dilution of formula. Thus, this study’s results reveal that many caregivers could benefit from interventions to improve formula-feeding practices, with the eventual beneficiary being the infants under their care.

Caregivers were not uniquely error-prone in this study, as even trained research assistants in highly controlled conditions made significant and potentially meaningful errors in measuring powder and water. Their careful measurements of compacted powder also demonstrated one way that the formula can be systematically under-diluted. For the non-compacted measurement conditions, the trained research assistant measurement standard values that were achieved by research assistants may estimate the best-case scenario for what is possible among caregivers. It is unlikely that caregivers could achieve such a consistently lower level of error without additional technological tools, such as the implementation of a high-precision scale during preparation. Clearly, the current tools provided in the form of powder formula, scoop, and bottles contribute to measurements being made that are rife with error. Additional factors, such as familiarity with the formula preparation products, may exacerbate those errors.

There is a notable scarcity of research on how best to help caregivers reduce errors while preparing infant formula. Research could address the lack of standardized guidelines for manufacturing baby bottles with precise measurement markings as well as better methods for measuring infant formula powder. Addressing these gaps should be a priority in research to mitigate some of the risks associated with formula feeding. Generally, future research on systematic error-reduction interventions is needed for what appears to be a persistent and widespread problem documented in the literature, particularly given that the problem of formula mismeasurement and under-dilution has potentially profound consequences for population health.

## Data availability statement

The raw data supporting the conclusions of this article will be made available by the authors, without undue reservation.

## Ethics statement

The study was conducted in accordance with the Declaration of Helsinki, and approved by the Institutional Review Board of the University of Nevada, Las Vegas (#UNLV-2022-464, November 2022) and by the Institutional Review Board of Kansas State University (IRB-11215, June 2022).

## Author contributions

RR: Conceptualization, Formal analysis, Funding acquisition, Methodology, Supervision, Writing – original draft. AG-A: Conceptualization, Data curation, Writing – review & editing. CA: Data curation, Writing – review & editing. AH: Formal analysis, Writing – review & editing. JH: Data curation, Writing – review & editing. CB-P: Data curation, Writing – review & editing. SR: Conceptualization, Methodology, Writing – review & editing.
