# Supplementary material for: Variability and error in measurement of infant formula powder and water: an experimental study
Source: Front Nutr. 2024 Aug 7;11:1385496. doi: 10.3389/fnut.2024.1385496 (PMC11335633; doi:10.3389/fnut.2024.1385496)
Supplement: Supplementary file 1 [file Table_1.DOCX]

Supplementary Material

# Supplementary Figures and Tables

## Supplementary Figures

|  | | | | | | | |
| --- | --- | --- | --- | --- | --- | --- | --- |
| 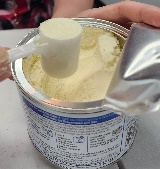 | 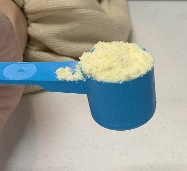 | 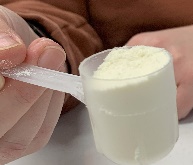 | 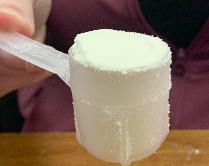 | 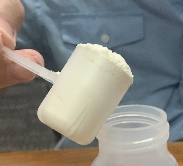 | 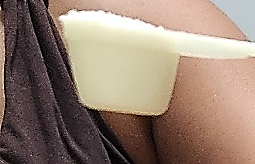 | 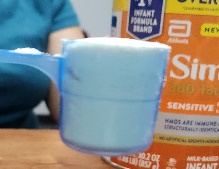 | 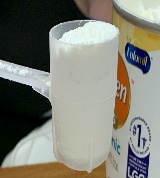 |
| 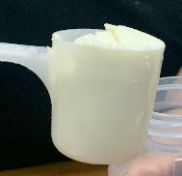 | 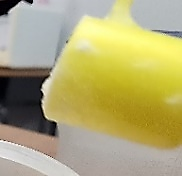 | 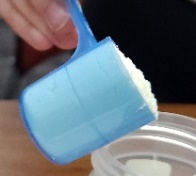 | 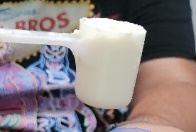 | 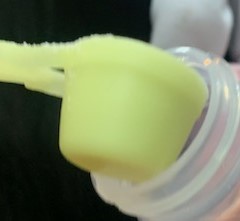 | 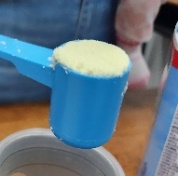 | 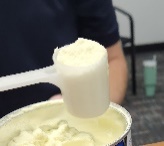 | 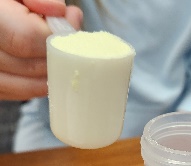 |
| 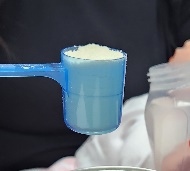 | 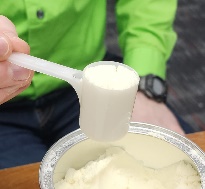 | 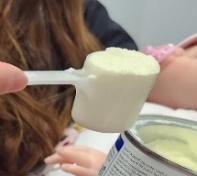 | 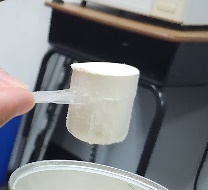 | 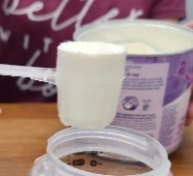 | 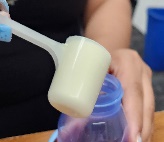 | 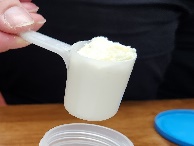 | 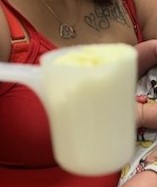 |
|  |  |  |  |  |  |  |  |
|  |  |  |  |  |  |  |  |
| 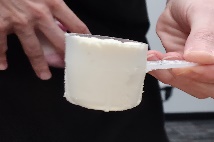 | 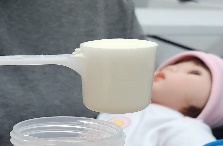 | 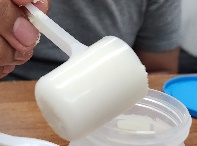 | 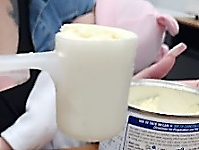 | 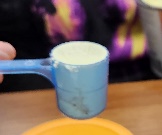 | 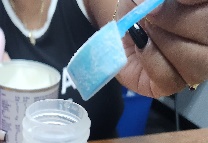 | 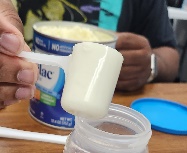 | 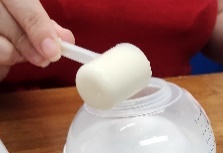 |
| 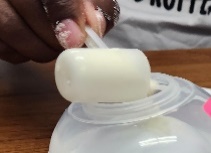 | 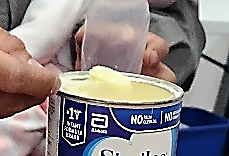 | 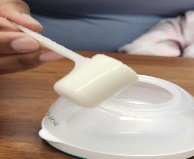 | 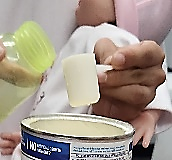 | 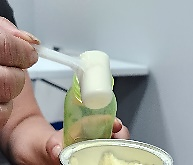 | 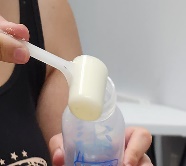 | 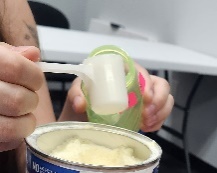 | 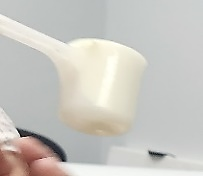 |
| 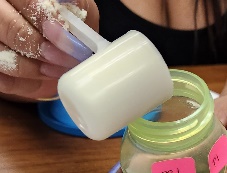 | 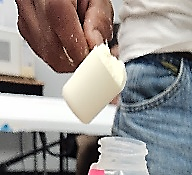 | 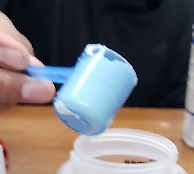 | 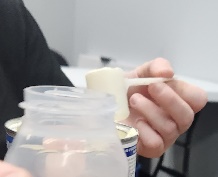 | 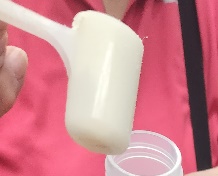 | 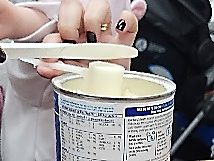 | 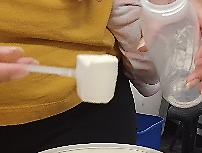 | 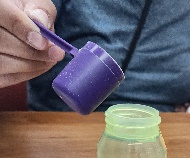 |
| 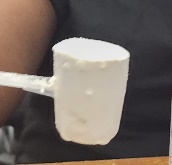 | 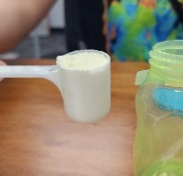 | 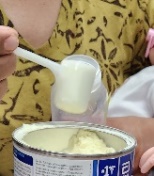 | 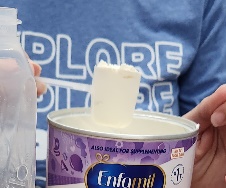 | 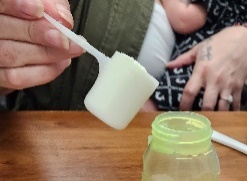 | 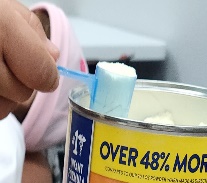 | 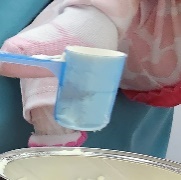 | 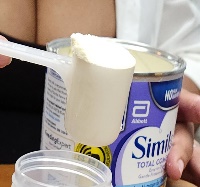 |
| 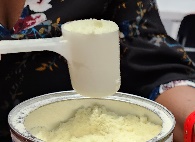 | 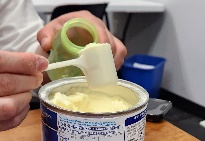 | 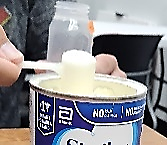 | 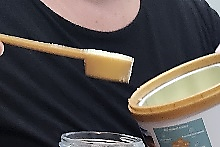 | 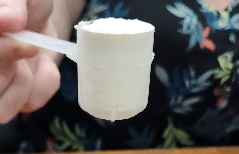 | 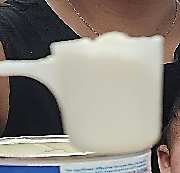 | 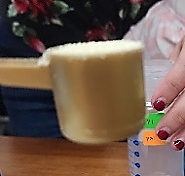 | 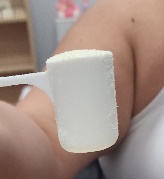 |
| 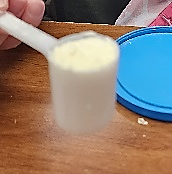 | 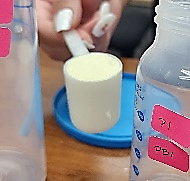 | 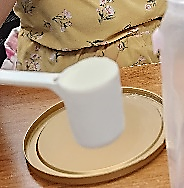 | 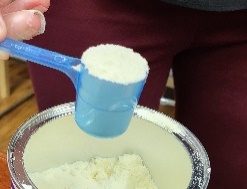 | 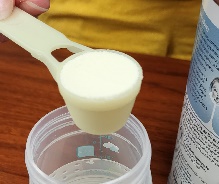 | 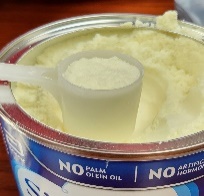 | 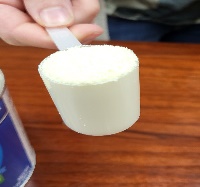 | 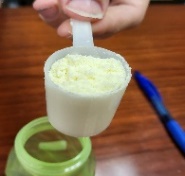 |
| 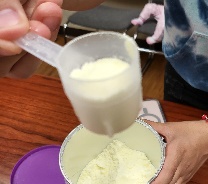 | 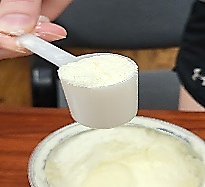 | 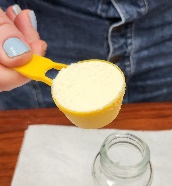 | 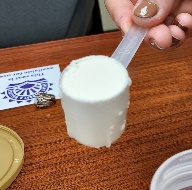 | 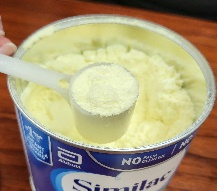 | 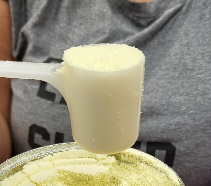 | 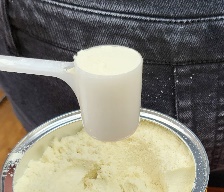 | 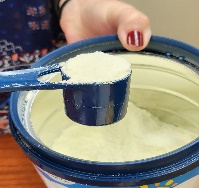 |
|  |  | 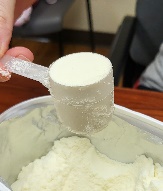 | 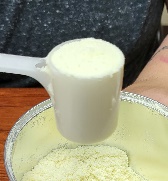 | 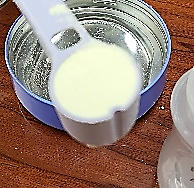 | 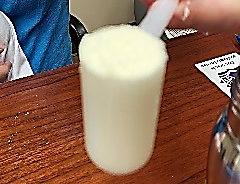 |  |  |

**Supplementary Figure 1.** Visual images portraying the variability in hand scooping of powdered formula
